# Draft Genome Sequence of *Pseudomonas aeruginosa* Strain WS136, a Highly Cytotoxic ExoS-Positive Wound Isolate Recovered from Pyoderma Gangrenosum

**DOI:** 10.1128/genomeA.00680-15

**Published:** 2015-07-02

**Authors:** Martin Arnold, Daniel Wibberg, Jochen Blom, Sarah Schatschneider, Anika Winkler, Yvonne Kutter, Christian Rückert, Andreas Albersmeier, Stefan Albaum, Alexander Goesmann, Sabine Zange, Jürgen Heesemann, Alfred Pühler, Michael Hogardt, Frank-Jörg Vorhölter

**Affiliations:** a Institut für Medizinische Mikrobiologie und Krankenhaushygiene, Universitätsklinikum der Johann-Wolfgang-Goethe-Universität, Frankfurt, Germany; b Max von Pettenkofer-Institut, Ludwig-Maximilians-Universität, München, Germany; c CeBiTec (Center for Biotechnology), Universität Bielefeld, Bielefeld, Germany; d Institut für Mikrobiologie der Bundeswehr, München, Germany

## Abstract

*Pseudomonas aeruginosa* is an opportunistic pathogen that typically infects patients with a compromised immune defense. Here, we present the improved 6.5-Mb draft genome of strain WS136, an ExoS-positive and ExoU-negative highly cytotoxic chronic wound isolate recovered from pyoderma gangrenosum of a patient who received bone marrow transplantation.

## GENOME ANNOUNCEMENT

*Pseudomonas aeruginosa* is an opportunistic Gram-negative gammaproteobacterium that is frequently implicated in a wide range of acute and chronic infections—especially infections of chronic wounds, such as pressure ulcers, diabetic ulcers, venous ulcers, and arterial ulcers—and thus represents a significant burden on patients and health care systems. Chronic infections due to *P. aeruginosa*, such as pneumonia in cystic fibrosis patients, are associated with the growth of bacteria within antibiotic-resistant biofilms and their adaptation to the respective niche (1). *P. aeruginosa* genomes range from 5.8 to 7.3 Mb that comprise a core genome consisting of more than 4,000 genes plus a variable accessory gene pool.

Here, we announce the draft genome sequence of *P. aeruginosa* WS136, isolated from pyoderma gangrenosum of a patient following bone marrow transplantation. It was selected among a set of more than 320 chronic wound isolates that were collected between 2006 and 2008 due to its outstanding phenotype. WS136 was phenotypically characterized as described previously (2) and was susceptible against all available antipseudomonal antibiotics except colistin. Using O antisera (BioRad, Munich, Germany), it was classified as serotype O3, produced almost no pyocyanin, had extraordinarily high elastase (LasB) activity, and hyperproduced and secreted the type III-dependent exotoxin S (ExoS) (3). This correlated well with its complete cell cytotoxicity against the mouse macrophage cell line J774.

To obtain a draft genome of this outstanding strain, we extracted genomic DNA to construct a paired-end library for shotgun sequencing with the Genome Sequencer FLX (GS FLX) system by means of the Titanium technology (Roche) as described recently (4–7). Standard protocols were followed per the manufacturer’s instructions. Assembly with the GS *de novo* Assembler software (Newbler) covered 209,590,411 bases from 954,897 aligned individual reads, among them 207,090 paired-end reads. The average size of the paired-end DNA fragments was 2,776 ± 694 bases. The assembly resulted in 92 contigs of at least 500 bp that were organized in 6 scaffolds by utilizing the paired-end information. An *in silico* gap closure approach (7, 8) reduced the contigs to 70. The scaffolds covered 6,548,669 bp, with an average coverage of 32×. The genome had a G+C content of 66.4%. Automated genome annotation was carried out by means of the GenDB software (9) and predicted 5,968 protein-coding sequences (CDSs) and 69 RNA-coding genes. Comparative analysis employing the EDGAR software (10) revealed the presence of a wild-type *exoS* gene, the absence of an *exoU* gene, and no significant mutations in the *lasB* gene or its 500-bp upstream region (3). A more detailed analysis of the genome will contribute to our understanding of wound-infecting pseudomonads and facilitate deeper insights into the combinatorial activity of virulence genes in *P. aeruginosa*.

### Nucleotide sequence accession numbers.

The whole-genome sequencing project for *P. aeruginosa* WS136 has been deposited at EMBL/GenBank/DDBJ under the accession number CBXZ000000000. Here, the first version (CBXZ000000000.1) of the genome data is described.
